# The CHOICE (Choice of Health Options In prevention of Cardiovascular Events) replication trial: study protocol

**DOI:** 10.1186/1471-2261-8-25

**Published:** 2008-10-06

**Authors:** Lis Neubeck, Julie Redfern, Tom Briffa, Adrian Bauman, David Hare, SB Freedman

**Affiliations:** 1 Faculty of Medicine, University of Sydney, Sydney, Australia; 2 ANZAC Research Institute, Concord Hospital, Sydney, Australia; 3 NHMRC NICS-Heart Foundation Fellow, Melbourne, Australia; 4 School of Population Health, University of Western Australia, Perth, Australia; 5 School of Public Health, University of Sydney, Sydney, Australia; 6 Department of Cardiology, Austin Health, Department of Medicine, University of Melbourne, Melbourne, Australia; 7 Department of Cardiology, Concord Repatriation General Hospital, Sydney, Australia

## Abstract

**Background:**

Although morbidity and mortality from coronary heart disease (CHD) are high, only a minority of acute coronary syndrome (ACS) survivors accesses an effective secondary prevention program. We aim to determine whether the previously proven CHOICE program can be replicated at multiple sites and whether ongoing reinforcement further improves risk factor modification.

**Methods/design:**

Participants eligible for but not accessing standard cardiac rehabilitation will be randomly allocated to either a previously tested 3-month CHOICE program or a 30-month CHOICE program (CHOICE-*plus*). Both groups will participate in individualised risk factor modules of differing duration that involve choice, goal setting and telephone follow-up for three months. CHOICE-*plus *will also receive additional face-to-face and telephone reinforcement between three and 30 months. At one site we will recruit a randomised control group, receiving conventional care. Primary outcomes are lipid levels, blood pressure, physical activity levels and smoking rates. Secondary outcomes include readmission rates, death, the number of risk factors, other modifiable risk factors, quality of life and process evaluation measures over three years.

**Discussion:**

We present the rationale and design of a multi-centre, replication study testing a modular approach for the secondary prevention of CHD following an ACS.

**Trial Registration:**

[Clinical Trial Registration Number, ACTRN12608000182392]

## Background

Coronary heart disease (CHD) is a leading cause of death and disability in Australia and costs associated with treatment are high [1]. Survivors of an acute coronary syndrome (ACS) have a 5% risk of a recurrence, some six times greater than that of the general population [2] and are advised to participate in a secondary prevention program [3,4]. Such programs incorporate lifestyle advice and pharmacotherapy to reduce recurrent cardiovascular events, improve survival and enhance quality of life [4]. The population is aging and thus the prevalence of CHD is expected to increase [5]. Similarly, the number of people surviving an ACS is rising largely due to improvements in acute treatment [6,7]. Because of these increases, the need to provide effective secondary prevention programs is escalating. In addition, three recent large studies concluded that effective risk factor reduction has reduced mortality from CVD by around 50% [6-8].

Cardiac rehabilitation (CR) programs aim to reduce coronary risk factors, facilitate patients return to normal activities and reduce their overall cardiovascular risk, in addition to stabilising, slowing or even reversing the underlying atherosclerotic process [9,10]. However, despite the potential benefits of CR, only a minority of people with CHD (10–30%) participate in existing programs [11,12]. Reasons for poor attendance include geographical location, being of lower socio-economic group and low referral rates for the elderly and women [13]. In addition, even in those who attend, long-term compliance with exercise-only CR regimens is only around 50% [13].

Disconcertingly, the majority of ACS survivors, who are at high risk of a future cardiac event, do not access a formal secondary prevention program resulting in a significant evidence-practice gap. In a single-centre study, it was demonstrated that ACS survivors not accessing CR had higher levels of individual risk factors including elevated total cholesterol (TC), low density lipoprotein (LDL) cholesterol, current smokers, hypertension, overweight, are less physically active, and were therefore at much greater overall risk than CR attendees [14]. Non-CR attendees also had much poorer knowledge of their risk factors, where 75% were unable to state even one of their own risk factors [14].

CHD is a chronic condition, and changes to lifestyle and adherence to pharmacotherapy have to be life-long. Studies have tried to address this evidence-practice gap through education and counselling of risk factor targets and medications with success [15,16]. Using a simple, innovative patient-centred modular approach to secondary prevention in a randomised controlled trial (n = 208) [17,18], it was found that the Choice of Health Options In prevention of Cardiovascular Events (CHOICE) program was readily acceptable to ACS survivors who did not access standard CR. It was also found that the intervention group significantly reduced individual cardiovascular risk factors and overall risk, and had significantly better risk factor knowledge at both three and 12 month follow-up compared to baseline and compared to having conventional care with their usual general practitioner and/or cardiologist [19-21]. Therefore, it was demonstrated at a single hospital site that the evidence-practice gap could be reduced for up to 12 months.

The original CHOICE trial provided a successful secondary prevention option for patients not accessing CR at a single site but it is now imperative to determine if the findings are replicable at multiple sites, delivered by multiple health professionals and to examine the processes which influence the implementation of a successful trial into a "real-life" setting [22]. The aim of this study is to establish if the CHOICE program can be replicated at multiple hospitals over three years. We also aim to investigate whether 30 months of telephone support is more beneficial than three months.

## Methods/design

### Design

**Figure 1:**
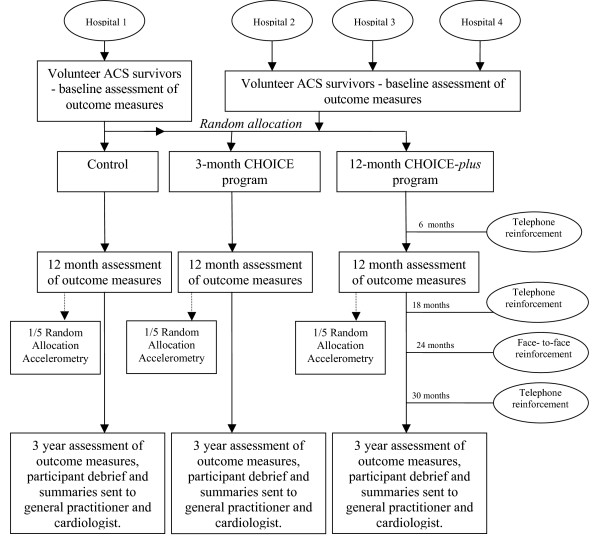
**Design of the CHOICE study**. ACS: acute coronary syndrome. CHOICE: Choice of Health Options In prevention of Cardiovascular Events.

This replication study, with a single site randomised control group, with three year follow up will be conducted at four tertiary referral hospitals in Sydney, Australia (figure 1) and is registered on the Australian and New Zealand Clinical Trials Registry (ACTRN12608000182392). Across all four sites patients not accessing standard CR will be randomly allocated to either a group participating in a three month CHOICE program or a 30 month CHOICE program (CHOICE-*plus*). At one site, patients not accessing CR will be allocated to one of three groups; the previously described intervention groups or a randomised control group participating in conventional care (figure 1), which will allow investigation of the effect of an additional nutrition module and a more tailored approach to depression.

Blinded assessments will be conducted at baseline and 36 months during a face-to-face interview. At 12 months, assessments will be completed by the intervention nurse and to ensure validity, a sample of 20 patients will have their measurements repeated by an independent assessor, blinded to group allocation. At follow-up assessments, additional data will be collected, including details of any unplanned hospital admissions, medications and doses and details of visits to the family physician, cardiologist and attendance at any community program pertaining to their heart health. Ethical approval for this study has been granted by Sydney South West Area Health Services and will be sought from the University of Sydney Human Research Ethics Committees. Written and informed consent will be obtained from all study participants prior to commencement.

### Study population

The hospital CR coordinators who review the Cardiology Department admission summaries daily will identify potential participants. Inclusion criteria for the study are; diagnosis of ACS up to eight weeks prior to recruitment; refusal of the initial invitation to participate in standard CR; failure to attend initial CR assessment. Exclusion criteria are: clinical diagnosis of uncompensated, severe cardiac failure (Class IV); uncontrolled arrhythmia or angina; severe or symptomatic aortic stenosis; persistent hypotension (SBP < 90 mmHg); clinical diagnosis of a severe coexisting medical condition that would prevent participation (e.g., dementia, a terminal illness, severe rheumatoid arthritis); or insufficient English to provide written informed consent. The primary criterion for eligibility for recruitment will be declining an offer to access existing CR. This will also include patients who are not offered CR while an inpatient but decline a subsequent offer to attend the CR program.

### Group allocation

Patients meeting the inclusion and exclusion criteria will be approached by the hospital CR coordinator or research assistant, during their inpatient stay where possible, or via letter or telephone soon after hospital discharge. Those who volunteer will have a baseline assessment and an initial face-to-face module selection and goal-setting session (approximately one hour in duration). This session will take place during the inpatient stay or in outpatient clinical consulting rooms, or, if necessary, at the participant's home, within one month of discharge. After baseline measurements are completed, at three sites, participants will be randomly allocated to one of the two groups (CHOICE or CHOICE-*plus*). At the remaining site participants will be randomly allocated to one of three groups (control, CHOICE or CHOICE-*plus*). Randomisation will be undertaken by an independent researcher (at the University of Sydney) with a computer-generated random allocation sequence and will be concealed from the health professional obtaining consent and conducting baseline assessment.

### Interventions

Participants randomly allocated to the control group at the single site will participate in ongoing conventional care, aimed at managing their cardiovascular health as directed by their family physician, ideally in consultation with their cardiologist. The control group will be advised to seek the advice of their family physician and cardiologist but will receive no additional intervention as a result of participation in the study.

Participants in both the CHOICE and CHOICE-*plus *groups will take part in a three-month patient-centred modular secondary prevention program. The CHOICE-*plus *group will also be offered additional face-to-face reinforcement at 12 and additional telephone reinforcement at six, 18 and 30 months.

**Figure 2:**
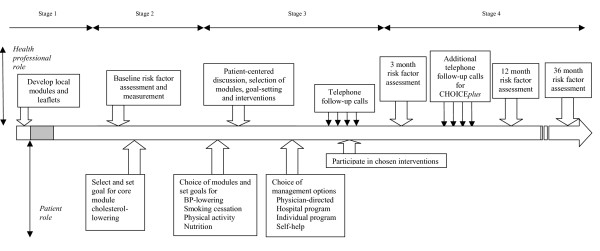
Model of CHOICE (Choice of Health Options In prevention of Cardiovascular Events) program after acute coronary syndrome (ACS).

As described elsewhere, the CHOICE intervention includes a one-hour initial consultation and follow-up phone calls over three months [20]. The program is designed to have an individualised, structured, case-management approach and is overseen by treating physicians. The development and implementation of the CHOICE program can be divided into four stages (figure 2). Stage one involves the development of modules and tailoring of leaflets to access recommended local services [21]. Stage two is a face-to-face risk factor assessment, lasting approximately one hour in which the patient is assisted to generate a list of their own relevant risk factors. In stage three, patients make guided choices about which risk factors they will address, participate in realistic goal-setting informed by national targets (table 1) [9] and then select management option(s) for lowering risk. The relevant patient information leaflets, described in Stage 1, support these choices. All CHOICE patients participate in the core module for lowering cholesterol as well as up to two choice modules from blood pressure (BP)-lowering, smoking cessation, increased physical activity and nutrition. Patients will have the option to add additional modules on completion of the three-month program. Additional module information will be given over the telephone and written information will be mailed to the patient. Stage four, is telephone follow-up, consisting of approximately four calls of around 10 minutes duration over a three-month period, during which each patient's risk factor(s) goals and strategies are re-evaluated and mutually changed if necessary. CHOICE-*plus *patients will receive additional telephone follow-up over 3 years as previously outlined (figure 1).

**Table 1:** Risk factor targets

**Risk factor**	**National Heart Foundation of Australia targets **[9]
Smoking	Complete cessation
Nutrition	Establish and maintain healthy eating
Alcohol	Low risk alcohol consumption
Physical activity	30 minutes of moderate activity on most days of the week
Healthy weight	Waist measurement
	Male ≤ 94 cm Female ≤ 80 cm
	BMI 18.5–24.9 kg/m^2^
Lipids	LDL < 2.0 mmol/l
	HDL > 1.0 mmol/l
	Triglycerides < 1.5 mmol/l
Blood pressure	Adults ≥ 65 (unless they have diabetes and/or renal insufficiency and/or proteinuria ≥ 0.25 g/day) <140/90 mmHg
	Adults <65 years; adults with diabetes and/or renal insufficiency and/or proteinuria 0.25–1.0 g/day <130/80 mmHg
	Adults with proteinuria >1 g/day <125/75 mmHg
Diabetes	Identify those with previously undiagnosed type 2 diabetes. In those with diabetes maintain HbA1C ≤ 7.0%

### Outcome measures

The primary outcomes are TC, systolic blood pressure (SBP), smoking rates and physical activity levels at 12 and 36 months (Table 2). Secondary outcomes include readmission rates, all-cause mortality, cardiac mortality, number of cardiac risk factors, waist circumference and process measures to assess barriers to implementation and fidelity to the intervention components (Table 2).

**Table 2:** Trial end points

**Primary**
TC (fasting blood sample)
SBP (resting digital reading) [27,28]
Smoking rates (self report confirmed with carbon monoxide meter) [29-31]
Physical activity (Active Australia Questionnaire validated by accelerometry) [24,26]
**Secondary**
Readmission rates (hospital records)
All-cause mortality (hospital records)
Cardiac mortality (hospital records)
Waist circumference (tape measurement) [32]
Number of modifiable risk factors (sum)
Process evaluation measures (including recruitment, withdrawal, barriers to participation, number and length of interventions delivered)
**Tertiary**
Diet (achievement of heart foundation target) [9]
Quality of life (SF12) [33]
Depression (Depression scale-short form) [34]
Angina status (Canadian Cardiovascular Society class) [35]
Modifiable risk factor knowledge (questionnaire) [19]
Cardioprotective medication use (hospital records and self-report)
Diabetic control (HbA1C)

Self-reported physical activity will be validated in a one-fifth subset of the cohort at 12 months by using accelerometers. Accelerometry is now considered the preferred method of objectively measuring physical activity as it provides data that allows individual examination of ambulatory activity frequency, intensity and duration [23,24]. For this trial an Actigraph GT1M (formerly Computer Science and Applications monitor), the most widely accepted accelerometer in research, will be utilised [25]. The matchbox size accelerometer will be attached to a belt that the participants in both the intervention and control groups will be asked to wear for a period of seven consecutive days at one year and three years. The Active Australia survey [26] records information from the seven-day period immediately prior to the survey, and therefore will be completed at the end of the week in which the accelerometer is worn. Thus the accelerometry data and Active Australia will record the same seven-day period.

To evaluate the generalisability of the previously proven CHOICE study [21], process measures of the intervention will include: record of participant recruitment, withdrawal from the program, the context of the interventions and the resources used. Barriers to implementation will be documented. Each participant will also answer questionnaires, which will determine the dose and exposure to the elements of the program. Through this we will examine fidelity of the program to the intervention components.

### Sample size

Given the compelling evidence that lowering TC lowers cardiovascular events, our sample size is calculated based on change in TC. In the previous study with 70/group, we found a 25.5 (SD 1.0) mg/dL (0.66 mmol/L) greater reduction in TC at 12 months in the CHOICE than control group. To demonstrate a difference between CHOICE and CHOICE-*plus *groups, 138 per group in the RCT will give 80% power (2-tailed, P < 0.05) to detect a 0.35 SD effect (13.5 mg/dl (0.35 mmol/L) difference) in TC at 12 months or 3 years. To demonstrate a difference between the control and intervention groups at the single randomized site, 64 patients will be needed in the control group for 80% power. To allow for 20% loss to follow-up and a design effect of up to 1.15 (ICC 0.05) due to cluster sampling, a total of 400 patients will be recruited across the four sites.

### Statistical analysis

Primary analyses will be conducted by intention-to-treat using SPSS for Windows (Version 12.01) and will be presented as mean and standard error of the mean or proportions. Differences in outcome measures, between and within groups, will be compared using repeated measures ANOVAs for continuous variables and either χ^2 ^tests or Fishers exact tests, as appropriate, for proportions of categorical variables. Two tailed p values of < 0.05 will be considered significant. Number needed to treat will also be calculated to estimate the number of patients who need to receive the CHOICE intervention to lower each of the major risk factors (TC, LDL-cholesterol, BP, smoking cessation, physical activity and overweight) to below current national targets.

## Discussion

The findings of this replication study could have wide-ranging implications for the management of patients with CHD who are at highest risk of adverse events, namely survivors of an ACS. We anticipate this study will demonstrate that the large evidence-practice gap for the many ACS survivors not accessing CR can be narrowed by a brief, flexible and tailored modular intervention (CHOICE) to change behavior, reduce overall cardiac risk, and thereby reduce the likelihood of death, infarction, and recurrent symptoms. By demonstrating the generalisability of the previously proven CHOICE program, this approach, using existing personnel and community resources could easily be adopted state-wide at relatively low cost.

The ultimate goal for our patients is to maintain behavioural change and risk factor reduction in the long term within their local community. At the conclusion of this study we anticipate that we will be able to determine whether ongoing reinforcement is important to lower risk, and if this strategy can be implemented at multiple sites and consequently narrow the evidence-practice gap in the longer term.

## Competing interests

The authors declare that they have no competing interests.

## Authors' contributions

LN drafted the manuscript and assisted with revision of study design and co-ordination. JR conceived of the study, design and co-ordination and helped to draft the manuscript. TB, AB, DH and BF were responsible for the original study design and review of the manuscript. All authors read and agree to the manuscript as written.

## Pre-publication history

The pre-publication history for this paper can be accessed here:


